# Granulocyte-macrophage colony stimulating factor (GM-CSF) enhances cumulus cell expansion in bovine oocytes

**DOI:** 10.1186/1477-7827-11-55

**Published:** 2013-06-24

**Authors:** Oscar A Peralta, Danai Bucher, Ana Fernandez, Marco Berland, Pablo Strobel, Alfredo Ramirez, Marcelo H Ratto, Ilona Concha

**Affiliations:** 1 Departamento de Fomento de la Producción Animal, Facultad de Ciencias Veterinarias y Pecuarias, Universidad de Chile, Santiago, Chile; 2 Institutos de Ciencia Animal y Bioquímica y Microbiología, Universidad Austral de Chile, Valdivia, Chile; 3 Facultad de Recursos Naturales, Universidad Católica de Temuco, Temuco, Chile

**Keywords:** GM-CSF, Oocyte maturation, Embryo development, GM-CSF receptors

## Abstract

**Background:**

The objectives of the study were to characterize the expression of the α- and β-subunits of granulocyte-macrophage colony stimulating factor (GM-CSF) receptor in bovine cumulus cells and oocytes and to determine the effect of exogenous GM-CSF on cumulus cells expansion, oocyte maturation, *IGF-2* transcript expression and subsequent competence for embryonic development.

**Methods:**

Cumulus-oocyte complexes (COC) were obtained by aspirating follicles 3- to 8-mm in diameter with an 18 G needle connected to a vacuum pump at −50 mmHg. Samples of cumulus cells and oocytes were used to detect GM- CSF receptor by immunofluorescence. A dose–response experiment was performed to estimate the effect of GM-CSF on cumulus cell expansion and nuclear/cytoplasmic maturation. Also, the effect of GM-CSF on *IGF-2* expression was evaluated in oocytes and cumulus cells after *in vitro* maturation by Q-PCR. Finally, a batch of COC was randomly assigned to *in vitro* maturation media consisting of: 1) synthetic oviductal fluid (SOF, n = 212); 2) synthetic oviductal fluid supplemented with 100 ng/ml of GM-CSF (SOF + GM-CSF, n = 224) or 3) tissue culture medium (TCM 199, n = 216) and then subsequently *in vitro* fertilized and cultured for 9 days.

**Results:**

Immunoreactivity for both α and β GM-CSF receptors was localized in the cytoplasm of both cumulus cells and oocytes. Oocytes *in vitro* matured either with 10 or 100 ng/ml of GM-CSF presented a higher (P < 0.05) cumulus cells expansion than that of the control group (0 ng/ml of GM-CSF). GM-CSF did not affect the proportion of oocytes in metaphase II, cortical granules dispersion and *IGF-2* expression. COC exposed to 100 ng/ml of GM-CSF during maturation did not display significant differences in terms of embryo cleavage rate (50.4% vs. 57.5%), blastocyst development at day 7 (31.9% vs. 28.7%) and at day 9 (17.4% vs. 17.9%) compared to untreated control (SOF alone, P = 0.2).

**Conclusions:**

GM-CSF enhanced cumulus cell expansion of *in vitro* matured bovine COC. However, GM-CSF did not increase oocyte nuclear or cytoplasmic maturation rates, *IGF-2* expression or subsequent embryonic development.

## Background

The granulocyte-macrophage colony stimulating factor (GM-CSF) is a glycoprotein with several molecular-weight species ranging from 18 to 30 kDa [1]. Its receptor is comprised of two cytokine-specific α-subunits and two signal transducing β-subunits [2]. Upon binding, the GM-CSF-receptor complex is capable of stimulating proliferation, maturation and viability of hematopoietic and non-hematopoietic cells [3,4]. The interaction of GM-CSF with its receptor stimulates multiple signal transduction pathways, including Jak/STAT pathway, Ras/Raf/mitogen-activated protein kinase pathway, phosphatidylinositol 3-kinase (PI 3-kinase)/protein kinase B (PKB) pathway, and protein kinase C (PKC) pathway [5]. GM-CSF promote glucose uptake through PI 3-kinase/PKB pathway via translocation of glucose transporter 1 (GLUT 1) [4]. GM-CSF induction of the PKB/Akt pathway results in direct cell survival activity and inactivation of proapoptotic factors BAD, caspase 9 and forkhead [6]. Additionally, Akt promotes cell survival indirectly by regulating a number of processes involved in glucose metabolism [6].

Both GM-CSF and its receptor have been highly characterized in the hematopoietic cell line, as well as in other cell types including fibroblasts, oligodendrocytes, trophoblast, endothelial and neoplastic [7-10]. In reproductive tissues, GM-CSF has been detected in testis, placenta, uterus, oviduct and ovary [11-15]. GM-CSF is expressed in utero by luminal and glandular epithelial cells and is subsequently secreted into the uterine lumen where activates neutrophils and macrophages during estrous cycle and early pregnancy [14,16]. The GM-CSF receptor has been detected from the fertilized oocyte through blastocysts stage in both mice and humans [17]. The selective expression of GM-CSF in theca, granulosa and lutheal cells coincides with peak follicular development, ovulation and luteinization [18-20]. In mice, cumulus-oocytes complexes (COC) express mRNA for the α-subunit of GM-CSF receptor, which has been reported to facilitates glucose uptake and thereby promote viability and proliferation in certain cell lineages [21]. Changes in estrogen and progesterone concentrations regulate the production of GM-CSF which suggests a potential regulatory function during the estrus cycle [14]. Thus, the expression of GM-CSF in the mouse ovary and uterus and its steroidogenic regulation suggest an autocrine/paracrine role in the ovarian physiology and embryonic development.

Oogenesis relies on the highly coordinated interaction between the oocyte and surrounding cells; the oocyte regulates follicular cell proliferation and differentiation and follicular cells control oocyte meiotic arrest [22,23]. Interaction of cumulus with the oocyte provides local production of glycosaminoglycans, steroid hormones, nutrients and other factors that support oocyte maturation [24-26]. Thereafter, presence of cumulus cells during IVF enhances fertilization and embryo development rates by facilitating sperm selection, capacitation, acrosome reaction and penetration [24]. Most of the energy required for these processes is supplied by glycolysis. However, glycolysis is limited during oogenesis due to reduced glucose transport and hexokinase activity in the oocyte [27]. *In vitro* studies have shown that cumulus cells are able to uptake and metabolize glucose allowing transport of glycolytic products such as pyruvate and lactate through gap junctions into the oocyte [28]. Pyruvate and lactate are easily oxidized by the oocyte becoming the main energy source during maturation [27,29]. Glucose might also be metabolized through the pentose-phosphate pathway (PPP) playing an important role in nucleotide biosynthesis and glutathione reduction during meiotic maturation and pronuclear formation [29]. Moreover, hyaluronic acid formation during cumulus expansion requires conversion of glucose into extracellular matrix components including glutamine [30]. Thus, the effect of GM-CSF on cumulus cells may potentially result in higher glucose uptake and cell proliferation or survival enhancing cumulus expansion. Alternatively, GM-CSF produced by macrophages within the ovarian stroma and theca cell layer may influence steroidogenesis and differentiation of thecal and follicular cells [20]. Taking these data together, we hypothesized that GM-CSF activity in the bovine COC may enhance oocyte maturation, cumulus expansion and subsequent embryonic development. To estimate the potential effect of GM-CSF at the transcription level, the expression of *IGF-2* may be quantified in bovine cumulus and oocytes after IVM. *IGF-2* is an imprinted gene in various mammal species and encodes an essential growth factor that plays a crucial role in tissue differentiation, fetal growth, and placental development [31]. In addition, *IGF-2* is believed to stimulate granulosa cells to produce estradiol, enhancing oocyte maturation [32].

The first objective of the current study was to characterize the expression of the α- and β-subunits of the GM- CSF receptor in bovine cumulus cells and oocytes. The second objective was to estimate the effect of exogenous GM-CSF on nuclear and cytoplasmic oocyte maturation, cumulus expansion, *IGF-2* transcript expression and subsequent competence for embryonic development.

## Methods

### Collection of oocytes and cumulus cells

All cell culture reagents were obtained from Sigma, unless otherwise specified. Bovine ovaries were obtained from a local abattoir and transported to the laboratory immersed in 0.85% saline supplemented with 100 mg/ml of Streptomycin and 80 mg/mL Sodium Penicillin G at a temperature of 35–38°C within 3 h of collection. Cumulus-oocyte complexes (COC) were obtained by aspirating follicles 3- to 8-mm in diameter with an 18 G needle connected to a vacuum pump at −50 mmHg. The follicular fluid was deposited in 60-ml tubes containing PBS-Dulbecco (8 mg/ml NaCl, 0.2 mg/ml KCl, 1.15 mg/ml KH_2_PO_4_, 0.10 mg/ml MgCl_2_ + 6H_2_O, 0.10 mg/ml CaCl_2_, 0.036 mg/ml sodium pyruvate, 1.00 mg/ml glucose) supplemented with BSA (3 mg/ml) and gentamicin (50 μg/ml).

### Immunofluorescence for GM-CSF detection in bovine oocytes and granulosa cells

Granulosa cells and oocytes were washed in 0.1 M PBS (pH 7.4, Gibco BRL) and fixed in a mixture of Histochoice and ethanol (4:1). Cumulus cells were obtained by vortexing COC for 5 minutes in PBS-0.1% BSA. Cells were then permeabilized and blocked in a solution of 0.1 M PBS with 1% BSA, 5% skim milk and 0.3% Triton X-100 for 60 min at room temperature. Cells were incubated in blocking solution (without Triton X-100) containing polyclonal antibodies (1:200; N-20 and C-18 for the GM-CSF alpha and beta subunit receptors respectively, Santa Cruz Biotechnology, California, USA) raised against the carboxyl and amino terminals of the α- and β-GM-CSF receptor subunits, respectively. After three washes with PBS, cells were incubated with anti-rabbit, anti-goat and anti-mouse IgGs (1:300 in blocking buffer) conjugated to Alexa Fluor 488 and 594 nm (Molecular Probes, California, USA), respectively. Cells were again washed three times in PBS and mounted under coverslips in a solution containing 4’, 6-diamidino-2-phenylindole (DAKO Laboratories, Denmark). Samples were examined under confocal microscope and photos were obtained using photomicroscopy (Olympus Fluoview 1000, Tokyo, Japan).

### In vitro maturation of cumulus oocyte complexes

After follicular aspiration, COC were classified into five groups based on the morphology of their surrounding cumulus cells [33]. Group A: with many layers of compact cumulus cells; Group B: with partially removed cumulus cells; Group C: denuded oocytes; Group D: degeneration of oocyte cytoplasm; Group E: expanded cumulus cells. Only COC classified as Group A and B were used in this study. Cumulus-oocyte complexes were then washed twice in PBS-Dulbecco (Gibco BRL) and twice in maturation media according to each treatment. Maturation media consisted of SOF (107.7 mM NaCL, 7.16 mM KCl, 1.19 mM KH_2_PO_4_, 1.5 mM D-glucose, 5 mM Taurine, 1.71 mM CaCL_2_, 0.49 mM MgCl_2_, 3.3 mM Sodium lactate and 25.07 mM NaHCO_3_) at pH 7.4 supplemented with aminoacids BME 50× (20 μl/ml), MEM 100x (10 μl/ml), BSA FV (8 mg/ml) and gentamicin (50 μg/ml). Cumulus oocyte complexes were randomly assigned to SOF medium supplemented with Recombinant human GM-CSF (hGM-CSF 215-GM-010 (R&D System, Inc., Minneapolis, USA) at concentrations of 1 (n = 71), 10 (n = 59) and 100 ng/ml (n = 89) [0.07, 0.7 and 7 nM, respectively]. Two additional groups were incorporated in the experimental design: SOF alone (n = 75) and a positive control maturation media consisted of tissue culture medium (n = 95) [TCM; 15 mg/ml TCM 199, 2.2 mg/ml NaHCO_3_ at pH 7.4] supplemented with 10% FBS (Hyclone, Utah, USA), 0.2 μM Pyruvate, 5 μg/ml LH (Lutropin, Bioniche, Belleville, Canada), 40 mg/ml FSH (Folltropin, Bioniche, Bellevile, Canada) and 50 μg/ml gentamicin.

Groups of 10–15 COC were allocated for *in vitro* maturation (IVM) in 50-μl droplets of treatment media in Petri dishes under mineral oil for 22 h in humidified atmosphere consisting in 5% CO_2_ at 38.5°C.

### Assessment of cumulus expansion and oocyte nuclear maturation

After 22 h of IVM, oocytes were collected and evaluated according to the cumulus expansion and then nuclear maturation. Cumulus expansion was determined using three different methods: 1) higher and a lower diameter for each COC were measured using a micrometric rule previously calibrated using a 0.1 mm objective (Nikon); 2) oocytes were microphotographed and higher and lower diameters were measured using a Fluoview software (FV 1000-ASW 1.4.3; Olympus, Corporation, Japan); and 3) a subjective scale was used [34] to estimate the degree of cumulus expansion. The degree of cumulus expansion was measured as follows: 0, no expansion; +1, separation of only the outermost layer of cumulus cells; + 2, further expansion involving the outer half of the cumulus oophorus; +3, further expansion up to, but not including, the corona radiate; +4, complete expansion, including the innermost corona radiate cells. A cumulus expansion index (CEI) [35] was calculated according to the subjective scale previously described using the following formula: CEI = (+1xn) + (+2xn) + (+3xn) + (+4xn) / N. Where CEI is the index for a given treatment, n is the total number of COC observed for each scale value in each treatment and N is the total number of COC in each treatment.

After cumulus expansion evaluation, cumulus cells were removed mechanically by vortex in PBS-0.1% BSA and washed twice in the same solution. Oocytes were placed between glass and cover slides with silicone and fixed with a mixture of acetic acid and ethanol (1:3) overnight at room temperature. Oocytes were then stained using 1% aceto-orcein for 1 h and destained using a mixture of acetic acid, glycerol and distilled water (1:1:3). Stained oocytes were examined under a phase contrast microscope for intact nucleus with germinal vesicle (GV), germinal vesicle breakdown (GVBD) or metaphase II (MII)-arrested.

### Determination of cumulus cell number and viability

Cumulus oocyte complexes (n = 52-60/per group) were randomly assigned to the following *in vitro maturation* media: SOF alone, SOF supplemented with GM-CSF at a concentration of 1, 10 or 100 ng/ml of GM-CSF or TCM 199 as described above. Groups of 10–15 COC were allocated for *in vitro* maturation (IVM) in 50-μl droplets of treatment media in Petri dishes under mineral oil for 22 h in humidified atmosphere consisting of 5% CO_2_ at 38.5°C. An additional sample of COC (n = 40-45/per group) was *in vitro* matured in SOF medium alone or supplemented with 10 and 100 μM of LY294002 a PI 3-kinase inhibitor or DMSO (DMSO was used as a diluent control). Cumulus cells were removed mechanically by vortex in PBS-0.1% BSA at 22 h. A 50 μl aliquot of cell suspension was mixed with 5 μl of Trypan Blue for cell viability using a Neubauer chamber.

### Assessment of oocyte cytoplasmic maturation

Cumulus oocyte complexes were randomly assigned to the following *in vitro maturation* media: 1- SOF without GM-CSF supplementation (n = 123), 2- SOF supplemented with 100 ng/ml of GM-CSF or 3- TCM 199 as described above (n = 159).

Immunohistochemical staining for cortical granules was also performed for evaluation of oocyte cytoplasm maturation. The type of cortical granules (type I, aggregates; type II, aggregates with some dispersion and type III, dispersion of granules) was evaluated as previously described [36]. Briefly, the zona pellucida was removed using 0.5% (w/v) pronase and oocytes were fixed in 4% (w/v) paraformaldehyde for 30 minutes. Oocytes were permeabilized with 0.25% Triton X-100 and washed with blocking solution (PBS containing 2% (w/v) BSA, 2% non-fat milk and 0.15 M glycine). Staining was performed using 10 mg/ml lens culinaris conjugated to fluorescein isothiocyanate (FITC, Sigma L9267, St Louis, USA).

Oocytes were examined and evaluated under epifluorescence inverted microscope (Nikon Corporation, Tokyo, Japan).

### Quantitative PCR

Relative expression of *IGF-2* gene transcript in bovine cumulus cells and oocytes were determined in COC (n = 30 per group) in *vitro* matured in TCM, SOF alone or supplemented with 100 ng/ml of GM-CSF. Total RNA was extracted from lysed cells using the RNAeasy extraction mini kit (Qiagen Inc., Valencia, CA, USA). All subsequent RNA purification steps were carried out according to the manufacturer’s instructions. cDNA was synthesized using the oligo-dT method (Promega Corp., Madison, WI, USA) with 1 μg of total RNA as a template in a reaction volume of 20 μl. Sequences of forward and reverse bovine *IGF-2* were: ATCCAGCCGCATAAACCG and GGACGGTACAGGGATTTCAG. A reaction mixture containing a volume of 50 μl was prepared (5 μl 10× PCR buffer, 2 μl dNTPs mix 10 mM, 2.5 μl forward and reverse primers 10 μM, molecular biology grade water and 0.5 μl Taq DNA polymerase). All the reagents were acquired from Promega. The reaction was heated on a Stratagene Thermo Cycler (GRI Systems, UK) to 95°C for 7 min, followed by 35 cycles of 94°C for 30 s, 55°C for 30 s, 72°C for 1 min, and a final extension step of 72°C for 10 min. As a normalization control for RNA loading, parallel reactions in the same multiwell plate were performed using *GAPDH* as a target. Quantification of gene amplification was made following quantitative PCR by determining the threshold cycle (C_T_) number for SYBR fluorescence within the geometric region of the semilog plot generated during PCR. Within this region of the amplification curve, each difference of one cycle is equivalent to a doubling of the amplified product of the PCR. The relative quantification of the target gene expression across treatment was evaluated using the comparative ∆∆C_T_ method. The C_T_ value was determined by subtracting the *GAPDH* C_T_ value from the target C_T_ value of the sample. Calculation of ∆∆ C_T_ involved using target gene expression on immature control (sample with the highest CT value or lowest target expression) as an arbitrary constant to subtract from all other C_T_ sample values. Relative target mRNA expression was calculated as fold changes in relation to immature control sample and expressed as 2^-∆∆CT^ value.

### In vitro fertilization and embryo development

A sample of COC was randomly assigned to *in vitro* maturation media consisting of: 1) SOF alone (SOF, n = 212); 2) SOF supplemented with 100 ng/ml of GM-CSF (SOF + GM-CSF, n = 224) or 3) Tissue Culture Medium (TCM 199, n = 216) and then subsequently *in vitro* fertilized and cultured for 9 days.

Embryos were produced using standard protocols for *in vitro* maturation, fertilization and culture [36-39]. Frozen-thawed semen from bulls of proven fertility (ABS, American Breeders Service, DeForest, WI, USA) was used for in vitro fertilization (IVF). The content of one 0.25-ml straw of frozen Holstein Friesian semen was thawed in water at 35–37°C. Thawed sperm were washed in a discontinuous gradient of 45/90% Percoll using centrifugation at 700 g for 20 min. The pellet was resuspended with washing medium TALP (Tyrode’s albumin lactate pyruvate) containing 6 mg/mL BSA (Fraction V), 1.0 mM Sodium Pyruvate and 5 μg/mL of gentamicin and centrifuged once again at 250 g for 5 minutes. After being centrifuged, the spermatozoa in pellets were counted and the volume adjusted to give a concentration of approximately 1.5-2 × 10^6^ sperm/ml of heparin-containing (1 μg/ml) TALP-IVF medium (TALP without glucose supplemented with 4 mg/ml BSA, 100 IU/ml penicillin and streptomycin, 0.1 mM pyruvate, and 2 μg/ml heparin). The sperm suspension was pippeted into 35 mm-petri dishes in 50 μl microdrops and covered with mineral oil. Thereafter, 10–12 matured COC per drop were added and incubated in 5% CO2 and 5% O2 in humidified air at 38.5°C. After 18–20 h, the presumptive zygotes were vortexed in PBS-0.1% BSA medium to remove the cumulus cells. Denuded zygotes were cultures in 30 μl of bicarbonate-buffered SOF medium for 7 days in a humid chamber under an atmosphere containing 5% CO_2_, 5% O_2_ and 90% N_2_.

### Embryo development and total cell number of blastocysts

Early cleavage was evaluated on Day 2 after *in vitro* fertilization (Day 0 = in vitro fertilization) and blastocyst formation were recorded on Days 7 and 9 of *in vitro* culture. Blastocysts from days 9 of *in vitro* culture (n = 30/per group) were used for cell number determination. Embryos were placed on a slide with dye bisbenzimide (Bis, Hoechst 33342, 10 μg/ml) for 5 min at 39°C. Hoechst dye was removed, and cover lips were mounted with wax and then firmly pushed onto the slide to spread the embryo. Staining nuclei was visualized with an epifluorescence microscope (Olympus, Tokyo, Japan).

### Statistical analysis

Single point measurements such as the difference among treatments for cumulus expansion, nuclear and cytoplasmic maturation, and *IGF-2* mRNA levels were estimated using one-way analysis of variance (ANOVA). Tukey’s multiple comparison was used as a post-hoc test when a significant difference was detected. Data from cumulus diameter was normalized to a logarithmic scale in order to accomplish homocedasticity. CEI and number of cells values were compared among treatments using non-parametric Kruskal-Wallis and multicomparison tests. Data from cellular viability were arcsin-transformed and analyzed using one-way ANOVA and Tukey’s test. All statistical analyses were performed using the Statistica 7.0 (StatSoft, Inc., Oklahoma, USA) software package.

## Results

### Expression of the GM-CSF receptor in bovine cumulus cells and oocytes

**Figure 1:**
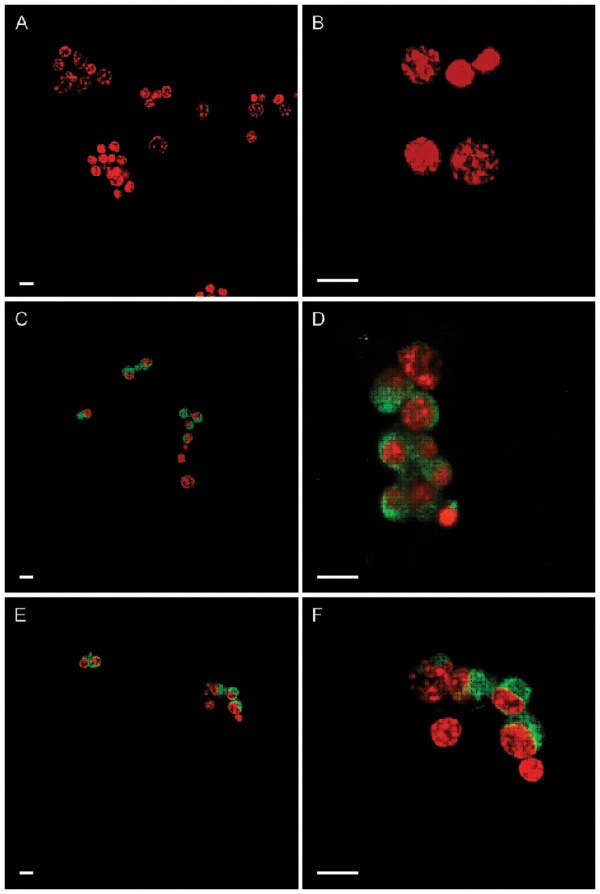
**Expression of GM-CSF α and β receptors in bovine cumulus cells.** Confocal microscopy analysis was performed using anti-GM-CSFrα and anti-GM-CSFrβ antibodies. **A**, **B:** Specimen incubated without the first (primary) antibody showed no signal. **C**, **D:** GM-CSFrα subunit (green). **E**, **F:** GM-CSFrβ subunit (green). Cell nuclei were stained using propidum iodide (red). Scale bar = 10 μm.

**Figure 2:**
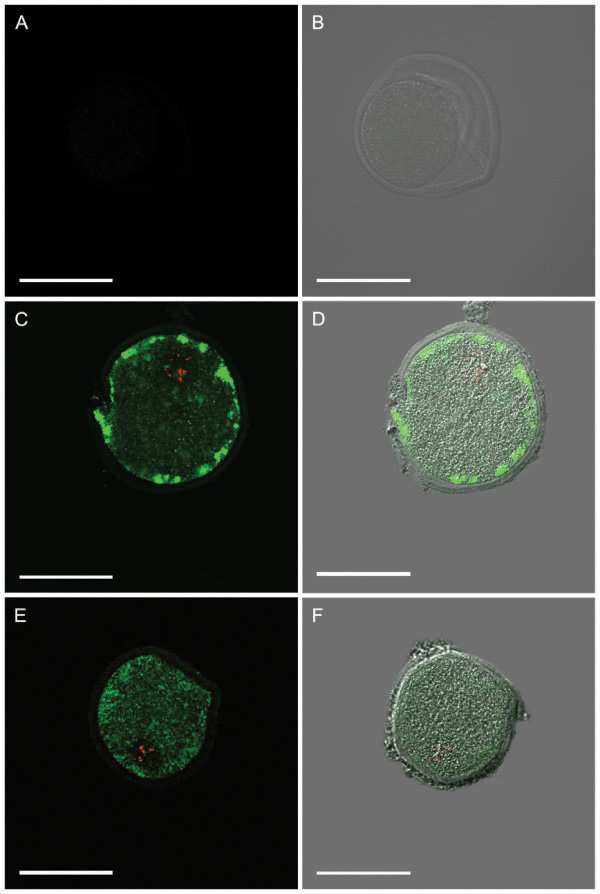
**Expression of GM-CSF α and β receptors in bovine COC.** Confocal microscopy analysis was performed using anti-GM-CSFrα and anti-GM-CSFrβ antibodies. **(A**, **B)** Specimen incubated without the first (primary) antibody showed no signal. **C**, **D:** GM-CSFrα subunit (green). **E**, **F:** GM-CSFrβ subunit (green). Oocyte nuclei were stained using propidum iodide (red). Scale bar = 100 μm.

Immunofluorescence analyses were performed to detect expression of α and β receptors of GM-CSF in whole-mounted bovine cumulus cells (Figure 1A-F) and oocytes (Figure 2A-F). Specimen (cells or oocytes) incubated without the first (primary) antibody showed no signal (Figures 1A,B; 2A,B).

### Effect of GM-CSF on the bovine oocyte in vitro maturation

A dose–response experiment was performed to estimate the effect of GM-CSF on nuclear maturation. The maturation state was evaluated using aceto-orcein staining. Data showed that the proportion of oocytes undergoing metaphase II after treatment with 1, 10 or 100 ng/ml of GM-CSF was not significantly different compared to the untreated controls (59.2, 54.2, 59.6 and 54.7%, respectively; Table 1). However, a higher proportion of metaphase II oocytes were found in the TCM treatment (83.2%).

**Table 1:** **Dose–response effect of the GM-CSF on nuclear maturation of bovine oocytes matured *in vitro ***

**Treatment**	**COC**	**GV**	**GVBD**	**MI**	**MII**
	**(n)**	**(%)**	**(%)**	**(%)**	**(%)**
*SOF*	75	11/78 (14.6)	9/75 (12.0)	14/75 (18.7)	41/75 (54.7)
*SOF + GM-CSF (1 ng/ml)*	71	11/71 (15.5)	5/71 (7.0)	13/71 (18.3)	42/71 (59.2)
*SOF + GM-CSF (10 ng/ml)*	59	8/59 (13.6)	4/59 (6.8)	15/59 (25.4)	32/59 (54.2)
*SOF + GM-CSF (100 ng/ml)*	89	14/89 (15.7)	4/89 (4.5)	18/89 (20.2)	53/89 (59.6)
*TCM*	95	1/95 (1.0)	4/95 (4.2)	11/95 (11.6)	79/95 (83.2*)

*GV* Germinal Vesicle, *GVBD* Geminal Vesicle Break-Down, *MI* metaphase I.

*MII* metaphase II. (*) Indicate significant differences between treatments (P < 0.05).

(There were 3 replicates for this experiment).

Further analyses were performed to estimate cytoplasmic maturation by cytoplasmic granule visualization using a fluorescence-labeled lectin. Treatment with 100 ng/ml of GM-CSF resulted in no significant differences in terms of percentage of type III oocytes compared to untreated or TCM controls (51.1, 55.3 and 53.5%; Table 2).

**Table 2:** **Effect of the GM-CSF factor on the type of cortical granules dispersion (cytoplasmic maturation) of *in vitro *** **matured bovine oocytes**

**Treatment**	**COC**	**Type I**	**Type II**	**Type III**
	**(n)**	**(%)**	**(%)**	**(%)**
*SOF*	123	37/123 (30.1)^a^	18/123 (14.6)^b^	68/123 (55.3)^c^
*SOF + GM-CSF (100 ng/ml)*	133	42/133 (31.6)^a^	23/133 (17.3)^b^	68/133 (51.1)^c^
TCM*	159	28/159 (17.6)^a^	46/159 (2.9)^b^	85/159 (53.5)^c^

^a,b,c^ Different superscripts within columns indicate significant difference (P < 0.05).

(There were 4 replicates for this experiment).

### Effect of GM-CSF on the bovine cumulus expansion and cell viability

**Figure 3:**
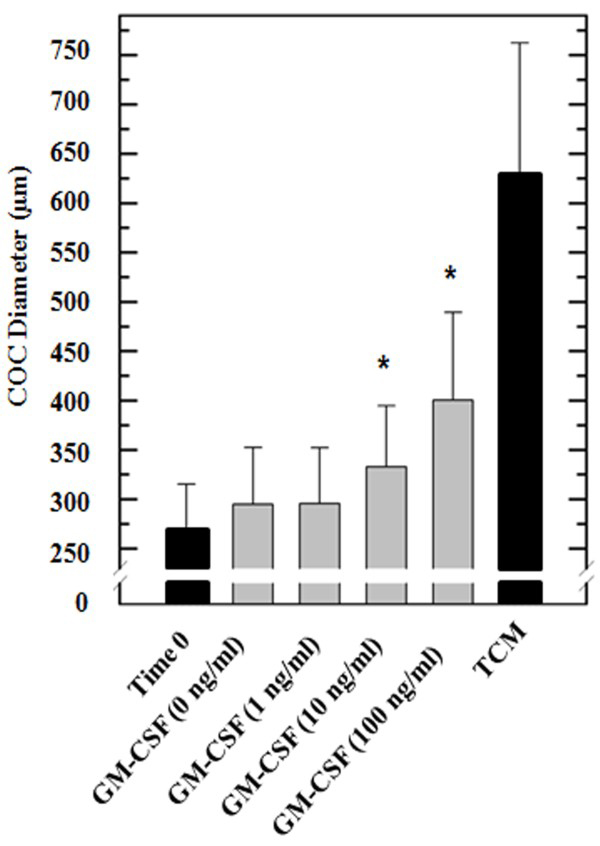
**Effect of GM-CSF on diameters of cumulus in *in vitro *matured COC. (A)** COC (n = 52-56/per group) were *in vitro* matured in SOF medium alone or supplemented with 1, 10 and 100 nM of GM-CSF. **(B)** Higher (P < 0.001) diameters were detected in COC matured in presence of 10 and 100 ng/ml of GM-CSF compared to untreated COC. Time 0 corresponds with the immature state of the oocytes. Parameters from oocytes in the immature state and matured in TCM were used as positive control and not included in the statistical analysis. (*) Indicate significant (P < 0.001) differences between treatments of 0, 1 and 100 ng/ml of GM-CSF.

**Figure 4:**
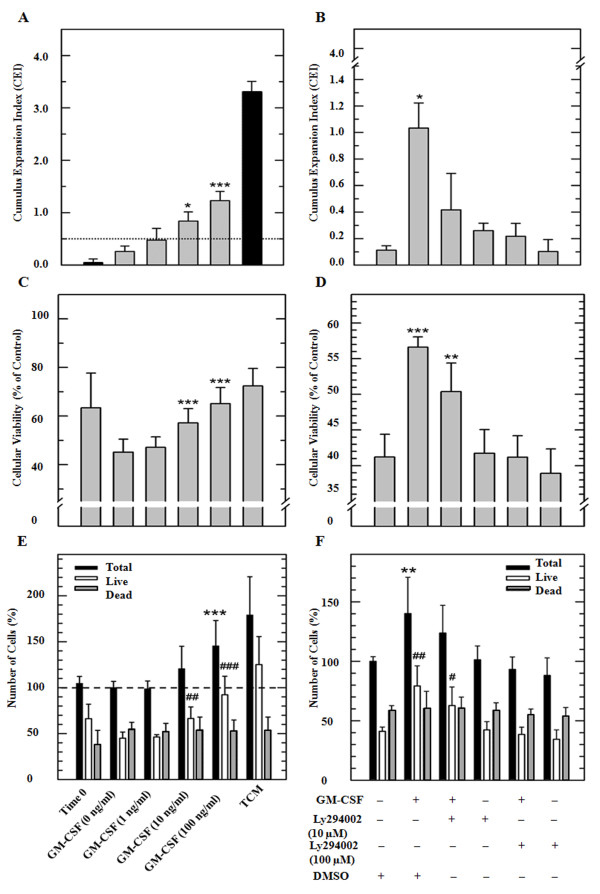
**Effect of GM-CSF on cumulus cell in *in vitro *matured COC.** (Left Graphs) COC were matured in SOF medium alone or supplemented with 1, 10 and 100 nM of GM-CSF. Parameters from cumulus cells derived from oocytes in the immature state and matured in TCM were used as positive control and not included in the statistical analysis. (Right Graphs) COC were matured in SOF medium supplemented with GM-CSF, LY294002 (10 or 100 μM) or DMSO. **(A**, **B)** Cumulus cell expansion was evaluated subjectively using the cumulus expansion index (CEI). **(C**, **D)** Cell viability was evaluated using Trypan Blue staining. **(E**, **F)** Cell number was determined using hemocytometer. (−−−) Corresponds with the lower limit for cumulus expansion (CEI ≥ 0.5). (*) Indicate significant differences between treatments of 0, 1 and 100 ng/ml of GM-CSF. *** P < 0.001, ** P < 0.01 and *P < 0.05. (#) Indicate significant differences compared with control ### P< 0.001, ## P< 0.01, # P < 0.05.

Cumulus expansion as an indirect indicator of oocyte maturation was estimated by calculating major diameters of cumulus and CEI before and after IVM. An increase (P < 0.001) in cumulus diameter was observed in COC treated with 10 and 100 ng/ml of GM-CSF (329 ± 68 and 400 ± 88 μm) compared with control COC (0 ng/ml, 295 ± 57 μm; Figure 3). Similarly, determination of CEI showed that both 10 and 100 ng/ml treatments induced higher (P < 0.05) cumulus expansion (0.85 and 1.22) after maturation compared to the untreated control (0.25; Figure 4A). To test whether GM-CSF has a direct effect on cumulus expansion, an inhibitor of the PI 3-kinase was added to the media. Addition of 10 or 100 μM of PI 3-kinase inhibitor to GM-CSF treated COC resulted in lower (P < 0.05) cumulus expansion (0.4 and 0.26) compared to COC matured only with GM-CSF (1.05; Figure 4B). The DMSO control showed no effect on cumulus expansion. After cumulus evaluation, cumulus cells were separated individually, stained using Trypan Blue and counted using a Neubauer chamber. Percentage of live cumulus cells decreased after *in vitro* maturation from 63.4 ± 14.3% to 45.2 ± 5.3% (Figure 4C). However, treatment with 10 and 100 ng/ml of GM-CSF resulted in higher (P < 0.001) percentage of live cells (57.2 ± 5.9 and 65.1 ±6.6%, respectively) compared to untreated cells (45.2 ± 5.3%). Treatment with 100 μM of PI 3-kinase inhibitor and GM-CSF resulted in lower (P < 0.001) percentage of live cells (41.1 ± 10.3%) compared to cells treated with GM-CSF alone (56.5 ± 13%) and cells treated with GM-CSF and 10 μM of PI3-kinase inhibitor (Figure 4D). To determine the potential effect of GM-CSF on cumulus cell proliferation, the total number of cells (live and dead) from all treatment groups were determined as described above (Figure 4E). Total cell number increased (P < 0.001) after the addition of 100 ng/ml of GM-CSF (147.4%) compared to untreated control (100%). Treatment with 10 and 100 ng/ml of GM-CSF resulted in more live cells (63.3%, P < 0.01 and 92.3%, P < 0.001, respectively) compared to the untreated control (44.5%). The addition of 100 μM of PI 3-kinase inhibitor in presence of GM-CSF resulted in lower number of total cells (94.1%, P < 0.01) and live cells (42.3%, P < 0.01) compared to cells treated with GM-CSF alone (140.2 ± 13% and 79.3%, Figure 4F).

**Figure 5:**
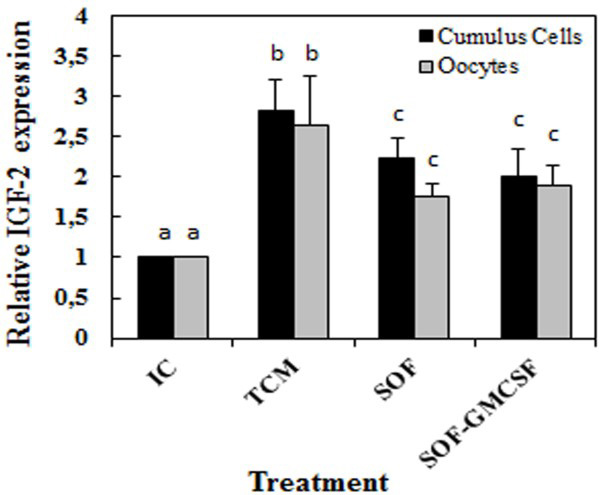
**Relative expression of *IGF-2 *gene transcript.** (mean ± SEM) in bovine cumulus cells (black bars) and oocytes (grey bars) in COC (n = 30/per group) *in vitro* matured in TCM, SOF alone or supplemented with 100 ng/ml of GM-CSF. (*) Indicate significant differences compared with immature control (IC). Different superscripts (a,b,c) indicate significant (P < 0.05) difference.

Furthermore, we evaluated the effect of GM-CSF during *in vitro* maturation on the mRNA *IGF-2* levels by Q-PCR. Relative *IGF-2* mRNA expression increased (P < 0.05) in cumulus cells and oocytes after in vitro maturation compared to the immature state. GM-CSF induced no significant effect over *IGF-2* expression neither in cumulus cells or oocytes (P > 0.05; Figure 5). However, *IGF-2* expression was up-regulated (P < 0.05) in cumulus cells and oocytes after maturation in TCM (2.81 and 2.64 fold compared to the immature control).

### Determination of the GM-CSF effect during oocyte maturation on subsequent embryo development

In order to evaluate the effect of GM-CSF *in vitro* maturation on subsequent embryonic development we utilized COC matured in TCM, SOF and SOF + 100 ng/ml of GM-CSF for in vitro fertilization. Results showed that COC exposed to 100 ng/ml of GM-CSF during maturation did not display significant differences in terms of embryo cleavage rate (50.4% vs. 57.5%), blastocyst development at day 7 (31.9% vs. 28.7%) and at day 9 (17.4% vs. 17.9%) or embryonic nuclei count (98 vs. 96) compared to untreated controls (P > 0.05; Table 3). However, oocytes matured in TCM showed higher (P < 0.05) cleavage and blastocyst development rates compared to oocytes matured in SOF and in SOF supplemented with 100 ng/ml of GM-CSF.

**Table 3:** **Fertilization and embryo development rates from bovine oocytes *in vitro *** **matured in TCM, SOF or SOF supplemented with 100 ng/ml of GM-CSF**

**Treatment**	**Oocytes (n)**	**Cleavage (%)**	**Blastocysts day 7 (%)**	**Blastocysts/ cleavage day 9 (%)**	**Blastocyst/ oocytes day 9 (%)**	**Embryonic nuclei (n)**
*TCM*	216	146 (67.6)^a^	55 (37.7)^a^	60 (41.1)^a^	27.8^a^	108.2 ± 7.7
*SOF*	212	122 (57.5)^b^	35 (28.7)^b^	38 (31.1)^b^	17.9^b^	98 ± 12.6
*SOF + GM-CSF (100 ng/ml)*	224	113 (50.4)^b^	36 (31.9)^b^	39 (34.5)^b^	17.4^b^	96 ± 13.1

^a,b^ Different superscripts within columns indicate significant differences (P < 0.05). (There were 4 replicates for this experiment).

## Discussion

Immunofluorescence analyses demonstrated a wide distribution of α- and β- subunits of the GM-CSF receptor in bovine oocytes and cumulus cells. Immunolabeling associated to both α and β receptors appeared to be located in the cytoplasm of cumulus cells. Oocytes collected from antral follicles were stripped from cumulus cells and processed for immunofluorescence analyses. Confocal microscopy showed a pattern of immunoreactivity for the α receptor in the cytoplasm in proximity to the plasmatic membrane. In contrast, the β subunit was homogeneously distributed in the cytoplasm. A previous report indicated the expression of the α-subunit but not the β- subunit in mouse COC by RT-PCR [20]. However, sections of mice and human ovarian tissue analyzed by in situ hybridization showed the transcript of both α- and β subunits as well as the GM-CSF ligand in the oocyte, theca, granulosa and luteal cells [18,19]. Moreover, RT-PCR analysis detected the expression of both subunits in human granulosa-lutein cell culture preparations [11]. Giving the abundant expression of GM-CSF receptor in bovine oocytes and granulosa cells, it is possible that this cytokine may play a significant role in the local regulation of the ovarian physiology. Evidence indicating a functional role of GM-CSF in reproduction has been provided by studies using GM-CSF knockout (GM-CSF −/−) mice. These animals exhibited longer estrous cycles, delayed blastocyst development, smaller litter size and higher rate of fetal death [40]. However, the biological role of GM-CSF has recently been associated to glucose transport in several non-hematopoietic cells including the spermatozoa [41]. GM-CSF increased glucose uptake via functional facilitative hexose transporters GLUT improving the freezing/thawing resistance and subsequent linear motility [12,41]. These finding, together with observations that GM-CSF and both subunits of the GM-CSF receptor are expressed in bovine oocytes and granulosa cells, suggest that GM-CSF may activate cumulus expansion and oocyte maturation, enhancing subsequent embryonic development. Our data demonstrates that supplementation of GM-CSF during *in vitro* maturation has no effect on the proportion of oocytes undergoing nuclear or cytoplasmic maturation. However, supplementation of GM-CSF induced higher cumulus expansion in *in vitro* matured bovine COC. Inhibition of the phosphatidylinositol 3 (PI3)-kinase prevented the GM-CSF effect, suggesting that the 3PI-kinase pathway is associated with glucose uptake, mediated by the activity of GM-CSF.

Addition of 100 ng/ml to cumulus cell culture resulted in higher percentage of total cells (45.4% higher) compared to untreated controls. The proportion of nonviable cells remained similar to controls indicating that a higher proportion of live cells accounted for the total cells. These data suggest that GM-CSF induced proliferation instead of survival of cumulus cells. The proliferative effect of GM-CSF was blocked after addition Ly294002 indicating that PI3- kinase activity mediated the GM-CSF effect. The intracellular signaling that intermediates proliferative and survival effects of GM-CSF have been previously characterized [42]. The proliferative effect is mediated by activation of major tyrosine phosphorylation-dependent signaling pathways including Jak/signal transducer and activator of transcription, Ras/mitogen-activated protein kinase, and PI3-kinase [43].

To estimate the effect of GM-CSF at the transcription level we quantified the expression of *IGF-2* in bovine cumulus and oocytes after IVM. Our results showed that *IGF-2* expression in cumulus cells and oocytes was not affected by GM-CSF treatment during in vitro maturation. However, we found that *IGF-2* was up-regulated in cumulus cells and oocytes after maturation. Moreover, *IGF-2* expression increased in oocytes and cumulus cells after maturation in TCM compared to maturation in SOF or supplementation with GM-CSF. Previous studies have suggested that *IGF-2* play an autocrine and paracrine role associated to survival activity in embryos cultured under suboptimal conditions [44]. These data suggest that *IGF-2* expression may be modulated by culture conditions but not by supplementation of GM-CSF.

We further tested the effect of GM-CSF on cumulus expansion and the potential effect on oocyte competence by evaluating the embryonic development of COC matured with GM-CSF. Cumulus expansion has been associated with several oocyte functions including ovulation, cleavage and embryonic development [24-26]. Cumulus cells also play an important role during fertilization by stimulating sperm selection and motility [25]. Using an *in vitro* system, oocytes treated with and without GM-CSF were fertilized with frozen/thawed semen and cultured in SOF for 9 days. We found no differences among treatments on cleavage rate, blastocyst development and embryonic nuclei count. These results indicate that the effect of GM-CSF on cumulus expansion during maturation was not sufficient to improve the subsequent embryonic development. Previous studies have showed that addition of GM-CSF to culture media improved development rates in bovine [45] and human [46] embryos. Moreover, exposure of bovine [47] and human [48] embryos to GM-CSF during development increased the percentage that developed to term.

## Conclusions

In conclusion, both α- and β-subunits of the GM- CSF receptor are expressed in bovine cumulus cells and oocytes. Despite, GM-CSF enhanced cumulus cell expansion of *in vitro* matured bovine COC, the oocyte nuclear and cytoplasmic maturation, *IGF-2* mRNA levels or subsequent competence for embryonic development was not affected by the GM-CSF treatment. Our data suggest that GM-CSF may play a role in cumulus cell expansion *in vitro* and increasing cell proliferation.

## Competing interest

The authors declare that they have not competing interest.

## Authors’ contributions

DB participated in designing the study, acquisition, analysis and interpretation of data, and in writing and revising the manuscript. OAP, AF, MOB participated in the acquisition and interpretation of the data. PS and AR participated in analysis and interpretation of data and critical revision of the manuscript. As Principal Investigators, MR and IC participated in the intellectual and experimental design of the study, the acquisition, analysis and interpretation of data, as well as writing and revising the manuscript. All authors read and approved the final manuscript.
